# Casein Glycomacropeptide Regulates Gene Expression in Intestinal Epithelial Cells: Effect of Simulated Gastrointestinal Digestion and Peptide Microencapsulation

**DOI:** 10.1021/acs.jafc.4c10146

**Published:** 2025-02-08

**Authors:** Raúl E. Cian, Mireia Tena-Garitaonaindia, Fermín Sánchez de Medina, Olga Martínez-Augustin

**Affiliations:** † Instituto de Tecnología de Alimentos, CONICET, FIQ-UNL, Santa Fe 3000, Argentina; ‡ Department of Biochemistry and Molecular Biology II, CIBERehd, School of Pharmacy, Instituto de Investigación, Biosanitaria (ibs.GRANADA), Instituto de Ciencia y Tecnología de los Alimentos José Mataix, 16741 University of Granada, Granada 18071, Spain; § Department of Pharmacology, CIBERehd, School of Pharmacy, Instituto de Investigación Biosanitaria (ibs.GRANADA), University of Granada, Granada 18071, Spain

**Keywords:** casein glycomacropeptide, intestinal epithelial cells, intestinal organoids, *in vitro* gastrointestinal digestion, dialyzability, encapsulation by spray drying

## Abstract

κ-Casein glycomacropeptide (GMP) exerts anti-inflammatory and immune modulatory effects. A bovine GMP concentrate and its *in vitro* digestion product were obtained. GMP was also microencapsulated with phycocolloids and further digested. These products were tested in three-dimensional (3D) and open monolayer two-dimensional (2D) mouse jejunal organoids. Almost no effect was observed on the 2D organoids. In 3D organoids, GMP induced intestinal proliferation (*Axin*2, *Pcna*) and differentiation (*Vil1, Alpl*) genes together with *Muc*3, antibacterial genes (*Lyz1, Pla2g2a*), and *Cxcl1*. GMP also induced interferon I defense genes (*Ifnb1, Ifr3, Oas2, Oas3, Rnasel*) under basal conditions and in TNF-stimulated organoids. *In vitro* digestion abrogated the effects of GMP and induced new genes (*Lgr5, Olfm4*, and *Lct*). In TNF-stimulated organoids, digested GMP repressed multiple genes. Encapsulation largely preserved the GMP effects. In conclusion, GMP showed differential effects in 3D and 2D organoids. The effects of digestion peptides were also different, suggesting distinct potential as functional foods.

## Graphical abstract

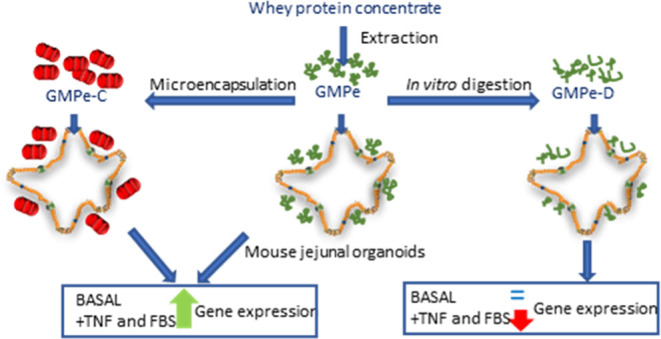

## 1 Introduction

Caseinmacropeptide or casein glycomacropeptide (GMP) is a bioactive peptide derived from κ-casein by the action of chymosin during cheese manufacturing and by the action of digestive enzymes during physiological digestion. It is, therefore, present in milk whey. GMP has proven under *in vitro* and *in vivo* conditions to exert a number of activities that regulate the physiology of important body systems, namely, the gastrointestinal, endocrine, and immune systems. This functional peptide is currently used in diets of people with liver illness because it is low in methionine and contains a lot of branched-chain amino acids. It is also appropriate for people with phenylketonuria because it does not contain phenylalanine.^1^ Other effects include the promotion of bowel movement and gall bladder contraction, and induction of the release of pancreatic enzymes.^1,2^

GMP has been shown to be anti-inflammatory in animal models of colitis, ileitis, and enteropathy.^3–11^ These effects have been related to prebiotic and protective effects against the intestinal microbiome.^12^ Immunoregulatory effects on specific cells have also been also described. Induction of Foxp3 in splenic cells from rats fed GMP for 3 days and in cultures stimulated with concanavalin has been described.^9^ Stimulation of cytokine production (TNF, IL-1β, and IL-8) in THP-1 cells (a monocyte/macrophage cell line) by GMP has been shown both in basal and lipopolysaccharide (LPS) stimulated conditions.^8,11^ This effect was exerted by intact GMP. In addition, when THP-1 cells were cultured in the presence of LPS the effect of GMP-derived peptides was the opposite, inhibiting cytokine production. These last observations indicated differential effects of intact GMP and its derived peptides.

Only a few studies have assessed the effect of GMP on the intestinal epithelium. These studies have used intestinal epithelial cell lines such as Caco-2 cells and HT-29 to mainly assess its anti-inflammatory effects,^13–15^ and its ability to inhibit the adhesion of a variety of pathogenic Escherichia coli strains, preserving the intestinal barrier function.^13^ Intestinal organoids are a much more complex system that allows a better characterization of the effect of products on processes of the intestinal epithelium that affect the intestinal barrier function such as cell differentiation and proliferation, immunity against bacteria and viruses, or the production of antibacterial peptides or mucins.^16^ Intestinal organoids are cultured from intestinal crypt cells that, after proliferation and in the presence of proper stimuli, differentiate, forming three-dimensional (3D) structures that contain several intestinal cell types, including enterocytes, Paneth cells, and goblet cells. Regular 3D organoids expose the basolateral side of epithelial cells to the culture media. This 3D organoid system has been widely used in the investigation of mechanisms for macronutrients and micronutrients affecting intestinal homeostasis.^17^ It may be useful to study the effect of luminal products in the case of intestinal damage. Nevertheless, several studies have shown that molecules with *M*_W_ ≤ 10.000 Da may cross the organoid barrier and therefore act from the luminal side. Organoid monolayers two-dimensional (2D) can be obtained from 3D organoids, where the effects of the addition of compounds to the luminal side can be studied directly. Here, we used both models to better assess GMP effects.

On the other hand, it was reported that gastrointestinal digestion degrades bioactive peptides. In this regard, several studies have demonstrated that most bioactive peptides containing more than 2–3 amino acid residues do not withstand simulated gastrointestinal digestion.^18^ However, the microencapsulation of bioactive peptides by spray drying using Pyropia columbinaphycocolloids as wall material efficiently protected the bioactive properties after simulated digestion.^19^ Thus, the use of this technology to protect GMP against digestive enzymes through encapsulation is a promising tool. Note that the spray drying technology produces a continuous matrix containing the active substances, enhancing the bioaccessibility of food-derived bioactive peptides.^18^ Moreover, the encapsulation of bioactive compounds in the food industry can be used to preserve functional properties, mask undesirable flavors, and increase the bioavailability in the food matrix.^20^

This work aims to better understand the effects of GMP and its derived peptides on intestinal epithelium. We obtained a GMP-enriched extract (GMPe) and used an *in vitro* model of gastrointestinal digestion to obtain GMPe-derived peptides (GMPe-D). In addition, GMPe was microencapsulated with phycocolloids from red seaweed P. columbina and maltodextrin in order to preserve the GMPe properties against digestive enzymes (GMPe-C). The effect of these products on the intestinal epithelium was studied using 3D organoids and monolayers from mouse jejunum organoids.

## 2 Materials and Methods

### 2.1 Raw Materials and Reagents

Whey protein concentrate (WPC) was provided by Milkaut (Franck, Santa Fe, Argentina). The moisture, protein, and fat contents of WPC were 3.26, 36.21, and 3.01 g 100 g^–1^, respectively. The carrier agents used for microencapsulation of bovine GMPe were phycocolloids (carrageenans and agars) and maltodextrin 15 DE (El Bahiense, Buenos Aires, Argentina). Phycocolloids were extracted from red seaweed P. columbina and maltodextrin according to Cian et al.^21^ Pepsin from porcine gastric mucosa (P-7000), and pancreatin from porcine pancreas (P-1750) were obtained from Sigma-Aldrich (St. Louis). Other reagents were of analytical grade and were obtained from Cicarelli Laboratorios (San Lorenzo, Santa Fe, Argentina).

### 2.2 Casein Glycomacropeptide-Enriched Extract

Casein glycomacropeptide was extracted from WPC according to Burns et al.^22^ Briefly, WPC was dispersed (20 g 100 g^–1^) in hot water (90 °C) at pH 5.0 for 20 min. The dispersion was centrifuged at 3500 rpm for 30 min at 55 °C (Cavour VT-3216, Buenos Aires, Argentina). The obtained supernatant was again centrifuged at 10,000 rpm for 30 min at 4 °C (Heal Force Neofuge 18R, Shanghai, China). Note that whey proteins are removed at this stage. The final supernatant was adjusted to pH 7.0 and ultrafiltered using a 5 kDa cutoff Molecular/Por Cellulose-Ester membrane and Molecular/Por Stirred Cell S-43–70 system. The volume reduction factor was 10. The fractions with molecular weight >5 kDa were lyophilized and named GMPe. The casein glycomacropeptide content from GMPe was determined by RP-HPLC using a casein glycomacropeptide standard (Sigma Chemical Co., St Louis, MO) according to Burns et al. For this purpose, a calibration curve of GMP was made (12–94 μg of GMP standard) and the peak areas were obtained at 7.546 min by RP-HPLC.

Fast protein liquid chromatography (FPLC) of WPC and GMPe was performed according to Cian et al.^23^ using an AKTA Prime system equipped with a Superdex 75 (GE Life Sciences, Piscataway, NJ). The molecular mass of protein fractions was estimated using molecular weight standards (Pharmacia Fine Chemicals, Piscataway, NJ). Moreover, the ratio between peak area and total area of chromatogram was performed.

Peptides analysis of GMPe was performed by RP-HPLC according to Garzón et al.^24^ For this, a Shimadzu LC-20AT pump, with an SPD-M20A diode detector and a Phenomenex C18 column (250 mm × 4.6 mm, 5 μm particle size) were used. Elution was performed by gradient, using water with 0.1% trifluoroacetic acid as the A mobile phase and acetonitrile with 0.1% trifluoroacetic acid as the B mobile phase. The gradient increased linearly, from 0 to 40% B, within the run time of 60 min. The column was placed at a temperature of 40 °C and the elution flow rate was 1 mL min^–1^. Fractions with absorbance peaks at 280 and 220 nm were analyzed. The retention times and areas of the peaks in GMPe were registered. Moreover, the ratio between peak area and total area of chromatogram was measured.

### 2.3 Microcapsules Formulation

For microencapsulation of GMPe, two formulations with phycocolloids from red seaweed P. columbina and maltodextrin as wall materials were made. The GMPe microcapsule (GMPe-C) formulation consisted of 1.7 g of phycocolloids from red seaweed P. columbina, 1.4 g of GMPe proteins (protein-to-wall material ratio 1:4), and maltodextrin to complete 10 g of total solids. Also, blank microcapsules without GMPe were produced (BC). Before the spray drying process, maltodextrin and GMPe were added to the phycocolloids dispersion (1.7 g 100 mL^–1^) and stirred for 1 h at 50 °C. 100 mL of 10 g 100 g^–1^ total solids dispersions were spray dried using a laboratory spray dryer (Mini Spray Dryer Büchi B-290, Büchi Labortechnik AG, Switzerland) equipped with an atomizer nozzle of 711 μm diameter, according to Cian et al.^25^ The airflow was 560 mL min^–1^ with an inlet temperature of 180 ± 4 °C and outlet temperature of 97 ± 1 °C. Pump pressure and feed flow were 2 bar and 5 mL min^–1^, respectively. The core/wall material ratio was 1:6.

### 2.4 Characterization of Microcapsules

The moisture and protein contents of microcapsules were determined using AOAC (2002) methods. The yield of microencapsulation process and encapsulation efficiency were determined according to Cian et al.^23^ and Garzón et al.,^19^ respectively. Encapsulation efficiency was defined as the amount of protein (GMPe) trapped in the core or surface of the carrier compared with the initial amount of GMPe in the formulation. All of the determinations were performed in triplicate.

The morphology and particle sizes of microcapsules were evaluated by scanning electron microscopy (SEM). Powders were mounted on aluminum stubs using double-sided tape and were coated with a thin gold layer using a cool sputter system (SCD 005, BAL–TEC, Switzerland). SEM images were acquired with a scanning electron microscope (SEM PHENOM PRO, Phenom World, The Netherlands) under high vacuum with a 20 kV acceleration voltage. Samples were observed with magnifications of 1000–8000×. SEM of each microcapsule was performed by triplicate. ImageJ (ImageJ, National Institutes of Health) was used to determine the microcapsule size.

Zeta potential of microcapsules was determined according to Cian et al.^25^ A dynamic light scattering and microelectrophoresis instrument Zetasizer Nano ZS90 (Malvern Instruments Ltd., Worcestershire, United Kingdom) with 633 nm He–Ne laser equipped with an MPT Autotitrator was used. Zeta potential was measured at pH 2.0 and 7.0. All determinations were performed in triplicate.

Surface hydrophobicity of GMPe-C and BC was determined according to Cian et al.^25^ using a Hitachi F-7000 fluorescence spectrophotometer (Japan). The slope of fluorescence intensity *vs* protein concentration (g 100 g^–1^) plot was calculated by linear regression analysis and used as an index of surface hydrophobicity. Moreover, the effect of NaCl and dodecylsulfate (SDS) on GMPe-C was evaluated according to Cian et al.^25^ For this, 10 mmol L^–1^ NaCl or 0.25% SDS solution was added to serial dilutions of microcapsules dispersions (0.05–0.005 g 100 g^–1^ protein). Then, measurements were carried out as mentioned before. Measurements were carried out in triplicate at room temperature.

### 2.5 Simulated Gastrointestinal Digestion and Bioaccessibility

In order to evaluate the effects of GMPe, GMPe-C, and BC on the regulation of gene expression in organoid culture after *in vitro* gastrointestinal digestion, the INFOGEST method described by Brodkorb et al.^26^ was used. The intestinal phase of the digestive process was simulated by using dialysis bags. After this process, bag contents corresponding to dialysates of the *in vitro* intestinal phase were transferred to flasks, weighed, and frozen at −20 °C until analysis. GMPe, GMPe-C, and BC dialysates from the gastrointestinal digestion process were named GMPe-D, GMPe-C–D, and BC-D, respectively. Protein in dialysates was measured according to Smith et al.’s method.^27^ Also, peptide analysis of GMPe-D and GMPe-C–D was performed by RP-HPLC as mentioned before.

### 2.6 Regulation of Gene Expression

#### 2.6.1 Organoid Culture and Treatments

Standard (3D) organoid culture was performed according to Arredondo-Amador et al.^28^ Briefly, jejunum crypts were cultured in a 1:1 dome of Matrigel Growth Factor Reduced Basement Membrane Matrix, Phenol Red-Free (Corning, New York) and IntestiCult Organoid Growth Medium (Mouse) (StemCell, Vancouver, Canada) following the manufacturer’s instructions. When Matrigel was set, 500 μL of IntestiCult were added to each well. After two to three organoid passages, experiments were conducted.

Organoid monolayers (2D) were generated from 3D organoids 5 days after seeding. 3D organoids were collected in cold PBS, shaken for 30 min at 50 rpm, and then centrifuged at 290 *g* for 5 min. The pellet was resuspended in TrypLE Express (Thermo Fisher Scientific, Alcobendas, Spain) and mechanically disrupted, followed by incubation at 37 °C for 5 min. The single-cell suspension was centrifuged at 600 *g* for 30 s and seeded in a Matrigel-coated 24-well plate using 10 μM ROCK inhibitor (Y-27632) and Primocin at 0.1 mg/mL in human IntestiCult (StemCell Technologies) for monolayer intestinal organoid culture. Experiments with organoids were authorized by the Committee on Animal Experimentation of the Junta of Andaluca (Spain, ref 17/02/2022/015).

To evaluate the regulation of gene expression, 2D and 3D organoids were treated with GMPe. In addition, 3D organoids were treated with GMPe-D or GMPe-C–D (0.1 g protein L^–1^) in basal and in tumor necrosis factor (TNF, 10 ng·ml^–1^) (eBioscences, San Diego, California) plus fetal bovine serum (FBS) 5% (v/v) stimulated condition. After 24 h of incubation, the organoids underwent RNA extraction.

#### 2.6.2 RNA Isolation and Quantitative Reverse-Transcription PCR (RT-qPCR) Analysis

Total RNA from tissue was isolated with the RNeasy minikit (Qiagen, CA), whereas organoid RNA was obtained with QIAzol Lysis Reagent (Qiagen, California), and 1 μg of RNA per sample was retrotranscribed using an iScript Select cDNA Synthesis kit (Bio-Rad Laboratories, California). Specific DNA sequences were amplified with a Bio-Rad CFX connect real-time PCR device (Alcobendas, Madrid, Spain). The primers used are shown in Table S1. Results are expressed as 2^–ddCt^ using *Ppib*, *Hprt*, and *18S* as reference genes.

### 2.7 Statistical Analysis

Each experiment was performed in triplicate. All results were expressed as mean ± SD (standard deviation). Statgraphics Centurion XV 15.2.06 software was used to analyze the data by analysis of variance (one-way ANOVA) and Duncan’s multiple range tests to determine differences between samples (*p* < 0.05). GraphPad Prism 6 software was used to analyze the RT-qPCR data. RT-qPCR data are given as relative expression (fold change) *versus* the control, which is assigned a mean value of 1. Differences among means were tested for statistical significance by analysis of variance (one-way ANOVA) and Fisher’s least significant difference tests to determine differences between samples (*p* < 0.05).

## 3 Results

### 3.1 Characterization of Casein Glycomacropeptide-Enriched Extract, Microencapsulation, and Simulated Gastrointestinal Digestion

Figure 1A shows the FPLC gel filtration profile of WPC and the casein glycomacropeptide-enriched extract (GMPe). WPC presented five main peaks of >75, 59, 20.3, 5.2, and <3 kDa. The components higher than 75 kDa presented an elution volume higher than that corresponding to the exclusion volume. This peak can be attributed to high-molecular-weight proteins such as immunoglobulins, lactoferrin, *etc.* The peak of 59 kDa corresponds to bovine serum albumin, while the 20.3 kDa peak corresponds to β-lactoglobulin and α-lactoalbumin. Similar results were reported by de la Fuente et al.^29^ for commercial WPC profile obtained with Superdex 75 column. The fourth peak between 4.6 and 7.8 kDa (5.9 kDa) corresponds to casein GMP. The 5.9 kDa peak area *vs* total area ratio was 2.4%, indicating that the proportion of GMP in the WPC was relatively low. Finally, the peak of <3 kDa could correspond to low-molecular-weight peptides.^29^ On the other hand, the GMPe profile presented two peaks of 7.3 kDa and <3 kDa. The first one corresponds, as noted, to GMP, while the <3 kDa peak corresponds to low-molecular-weight peptides. Note that the theoretical molecular weight of GMP is ∼8.0 kDa.^30^ Moreover, the GMPe profile does not show the characteristic peaks of whey proteins. In addition, the 7.3 kDa peak area with respect to the total area was 84%. These results indicate that the proportion of casein glycomacropeptide in GMPe was significantly and substantially higher than that found in WPC. Thus, the thermal process followed by ultrafiltration to obtain GMPe was appropriate. In this regard, the casein glycomacropeptide purity in GMPe was 71.4 ± 2.1 g × 100 g^–1^ protein.

**Figure 1:**
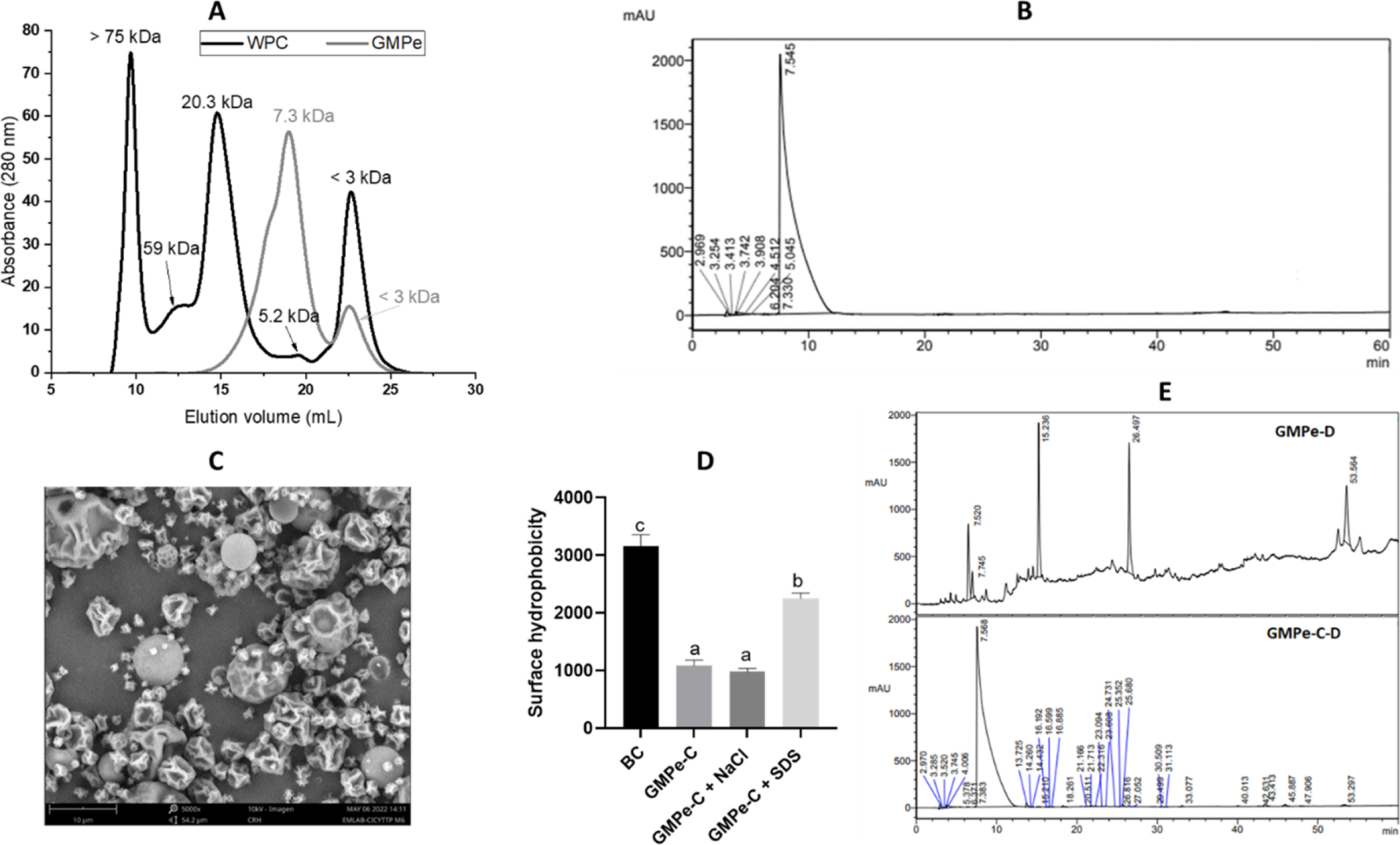
FPLC profiles of WPC and GMPe (A); RP-HPLC profile of GMPe (B); scanning electron micrographs of GMPe-C (C); surface hydrophobicity of BC, GMPe-C, GMPe-C + NaCl (10 mmol L^–1^) and GMPe-C + SDS (0.25%) (D); and RP-HPLC profiles of GMPe-D and GMPe-C–D (E). Different letters mean significant differences between samples (*P* < 0.05).

Figure 1B shows the RP-HPLC profile of GMPe. The peak at the retention time of 7.545 min corresponds to the casein glycomacropeptide. The additional minor peaks observed between 2.969 and 5.045 min could correspond to impurities. However, the ratio of the 7.545 min peak area *vs* the total area was 98.9% (*i.e.*, 1.1% for the other peaks). Thus, GMPe was greatly enriched with casein glycomacropeptide. These results agree with those reported by Barone et al. for RP-HPLC profile of casein glycomacropeptide obtained from WPC.^30^ These authors, using the same mobile phase and similar mobile phase gradient, found that GMP was detected at a retention time of 5.00 to 7.50 min. Moreover, different authors have reported that the HPLC retention time of GMP is lower than 10 min.^31–33^

Regarding the microencapsulation of GMPe, it was observed that the yield of the spray drying process for GMPe-C was 62.9 ± 0.7%. A similar result was reported by Cian et al.^23^ for Phaseolus lunatus peptides microencapsulated with maltodextrin and gum arabic at the same core/wall material ratio (1:6). On the other hand, the encapsulation efficiency of GMPe-C was 82.3 ± 2.9%. This high encapsulation efficiency value could be due to the wall material used. It was reported that the addition of phycocolloids from red seaweed P. columbina to microcapsule formulation increases encapsulation efficiency, mainly due to the good film forming properties of carrageenan and agar provided by this red seaweed.^21,25^

The surface morphology of GMPe-C is shown in Figure 1C. GMPe-C presented various sizes and shapes (spherical, irregular, and shrunk). Some microcapsules showed surface dents (indentations), which are typical of atomized products.^34^ The appearance of concavities on the surface of the particles obtained by spray drying is common and may be due to the rapid evaporation of liquid droplets during the drying process.^35^ It is important to note that no fissures, cracks, or disruptions were observed in the surface morphology of microcapsules. This is fundamental for guaranteeing higher protection and peptide retention.^19^ On the other hand, the mean apparent diameters of microcapsules—estimated from the SEM images—was 7.7 μm. However, wide size distributions were observed (from 0.9 to 25 μm). Similar results were reported by Cian et al.^25^ for brewers’ spent grain peptides microencapsulated with P. columbina phycocolloids (7 μm).

As shown in Figure 1D, GMPe-C had a lower fluorescence intensity value than microcapsules without GMPe (BC). This result suggests that during encapsulation, hydrophobic interactions occur between GMPe-C and P. columbina phycocolloids, decreasing the surface exposure of hydrophobic groups. Moreover, the addition of SDS increased the fluorescence intensity of GMPe-C, which can be due to the disruption of the hydrophobic interactions between GMPe and P. columbina phycocolloids. As it is known, SDS is a powerful disruptor of hydrophobic interactions among molecules.^36^ However, there were no changes in surface hydrophobicity between GMPe-C and GMPe-C + NaCl (*P* > 0.05), indicating that the interaction between GMPe and phycocolloids is not electrostatic. Similar results were reported by Cian et al.^25^ for brewers’ spent grain peptides microencapsulated with P. columbina phycocolloids.

The zeta potential values of GMPe-C at pH 2.0 and 7.0 were −13.8 ± 0.9 and −31.6 ± 0.3 mV, respectively. Garzón et al.^19^ reported that a zeta potential of at least ±8–9 mV at pH 2.0 confers stability to the microcapsules in the gastric tract. Thus, the value obtained for GMPe-C indicates that microcapsules could be potentially stable through the gastric environment. Moreover, GMPe-C had a strong negative charge at pH 7.0. In this regard, it was reported that zeta potential values of ±30 mV are required for a physical stable suspension.^34^ Thus, the GMPe-C suspension at pH 7.0 could be considered stable, with a low tendency to form particle aggregates.

Regarding the effect of simulated gastrointestinal digestion on GMPe and GMPe-C, it was observed that microencapsulation with phycocolloids from P. columbina largely preserved the GMP structure (Figure 1E). In this regard, the RP-HPLC profile of GMPe-CD showed a main peak at the retention time of 7.568 min that corresponds to casein glycomacropeptide. Moreover, the 7.545 min peak area:total area ratio was 97.0%. The presence of other peaks of smaller areas would be related to a small degradation of the GMPe by gastrointestinal proteases. Note that the total area of the other peaks amounted to 3.0% of the total area. In contrast, a notable degradation of casein glycomacropeptide by gastrointestinal proteases was observed in GMP-D, which is demonstrated by the reduction of the peak area at 7.5 min. In this regard, the 7.520 min peak area of GMP-D represents only 15% of the total area of chromatogram. In addition, a main peak is observed at a retention time of 26 min, corresponding to 30% of the total area. Therefore, the microencapsulation of GMPe with phycocolloids was a good strategy to preserve the structure of casein glycomacropeptide in the simulated gastrointestinal environment.

### 3.2 Addition of Bovine Glycomacropeptide to 3D Organoids Modulates Intestinal Barrier Function Gene Expression

Our first goal was to study the effects of GMPe on 2D and 3D organoids. GMPe was added for 24 h to organoid cultures, and qRT-PCR was performed. A set of genes related to proliferation, differentiation, and immune function was studied. While GMPe induced the expression of proliferation and differentiation markers in 3D organoids, no effects were observed in organoid monolayers (Figure 2). In fact, our results show that intact GMPe added to 3D organoids induced the expression of proliferation (*Pcna* and *Axin2*) and specific lineage cell markers (*Vil1*—encoding villin and *Alpl*—tissue nonspecific alkaline phosphatase).

**Figure 2:**
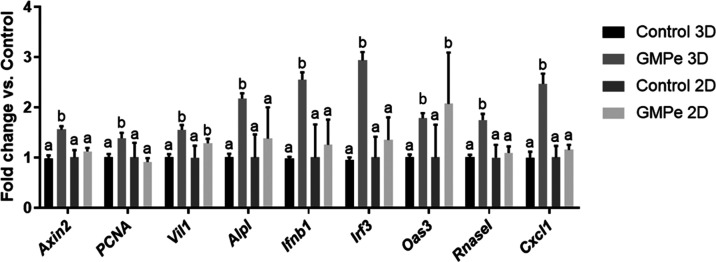
Expression of genes involved in the intestinal barrier function y mouse jejunum 3D and 2D organoids measured by different letters indicate different values *p* < 0.05. Data are representative of two different experiments, *n* = 4.

Regarding immune function, GMPe added to 3D organoids induced the expression of the proinflammatory cytokine *Cxcl1* and of antiviral response genes (*Ifr3*, *Ifn1b, Oas 3*, and *Rnasel*), while only *Oas3* was induced in 2D organoids.

Taking into account the results mentioned above and the fact that organoid monolayers did not respond to TNF (data not shown), we decided to continue our experiments using only 3D organoids with an expanded gene set. GMPe additionally induced the expression of *Lyz1, Pl2g2a, Muc3* (Figure 3) and *Oas2, Oas3*, and *RNasel* (Figure 4). TNF induced the expression of intestinal stem cell markers *Lgr5* and *Ang4*, while the expression of *Pcna* was inhibited (Figure 5). Addition of GMPe to the cell culture media of TNF-stimulated organoids partially abrogated the effect of TNF on *Lgr5* and induced *Pcna* expression, but did not affect *Ang4*. Inhibition of *Olfm4* by GMPe was observed in both basal and TNF-stimulated conditions (Figure 5).

**Figure 3:**
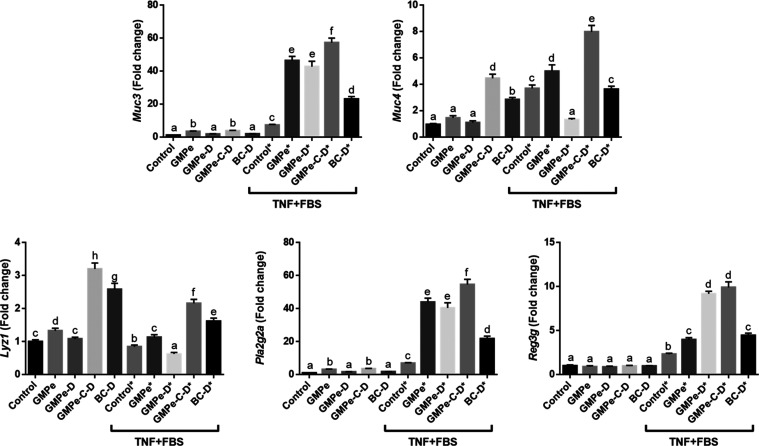
Expression of goblet and Paneth cell markers in 3D mouse jejunum organoids measured by qRT-PCR. Different letters indicate different values *p* < 0.05. Data are representative of two different experiments, *n* = 4.

**Figure 4:**
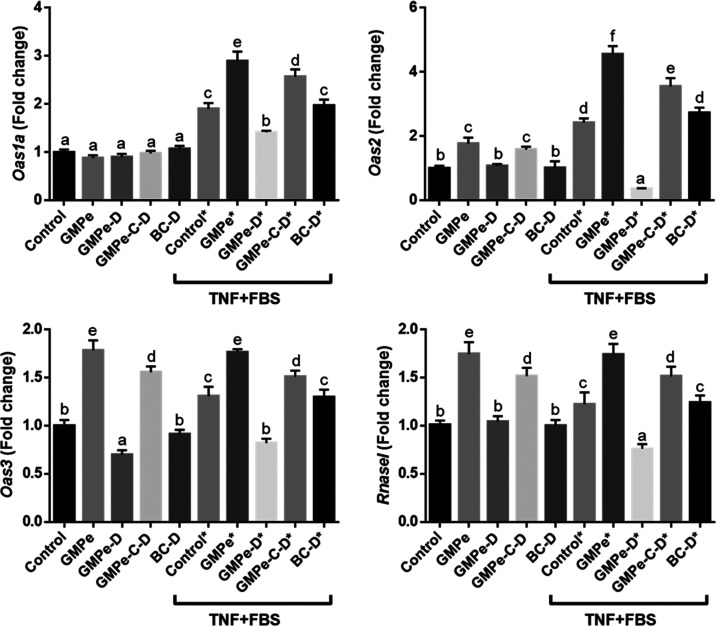
Expression interferon response genes in mouse jejunum 3D organoids measured by qRT-PCR. Different letters indicate different values *p* < 0.05. Data are representative of two different experiments, *n* = 4.

**Figure 5:**
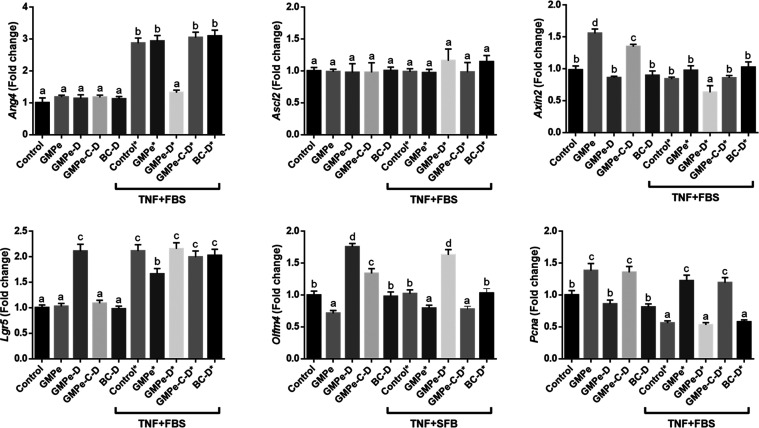
Expression of proliferation-related genes in mouse jejunum 3D organoids measured by qRT-PCR. Different letters indicate different values *p* < 0.05. Data are representative of two different experiments, *n* = 4.

Lineage markers and defense-related genes were modified by TNF. Namely, this cytokine induced the expression of the alkaline phosphatase isoforms, *Alpi* and *Alpl*, the mucins *Muc3* and *Muc4*, and the antimicrobial peptides *Pla2g2a* and *Reg3g*, while it downregulated the enterocyte differentiation markers *Lct* and *Sis*, plus *Lyz1* (Figures 3 and 6). In the presence of TNF, GMPe further induced the expression of *Muc3*, *Muc4*, *Reg3g* and *Pla2g2a*. It also counteracted the effect of TNF on *Sis* and *Lyz1* and further reduced the level of *Lct* expression (Figures 3 and 6).

**Figure 6:**
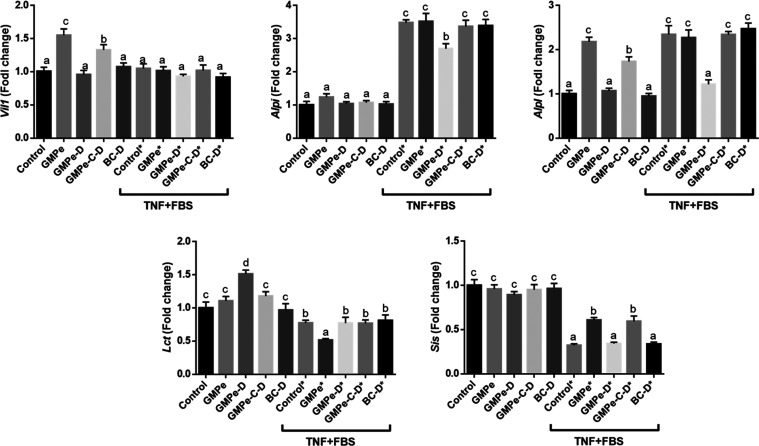
Expression of enterocyte genes in mouse jejunum 3D organoids measured by qRT-PCR. Different letters indicate different values *p* < 0.05. Data are representative of two different experiments, *n* = 4.

As expected, TNF induced the expression of proinflammatory cytokine *Cxcl1* and of some genes related to interferon-mediated antimicrobial response (*Ifit1*, *Ifn1b*, *Oas1a*, *Oas2*, *Oas3*, and *Rnasel*) (Figures 4 and 7). The stimulation of the antiviral and inflammatory response was enhanced by the addition of GMPe to the culture medium, resulting in further induction of *Cxcl1*, *Oas1a*, *Oas2*, *Oas3*, and *Rnasel*, and induced the expression of *Ifr3*, although much less than in the absence of TNF.

**Figure 7:**
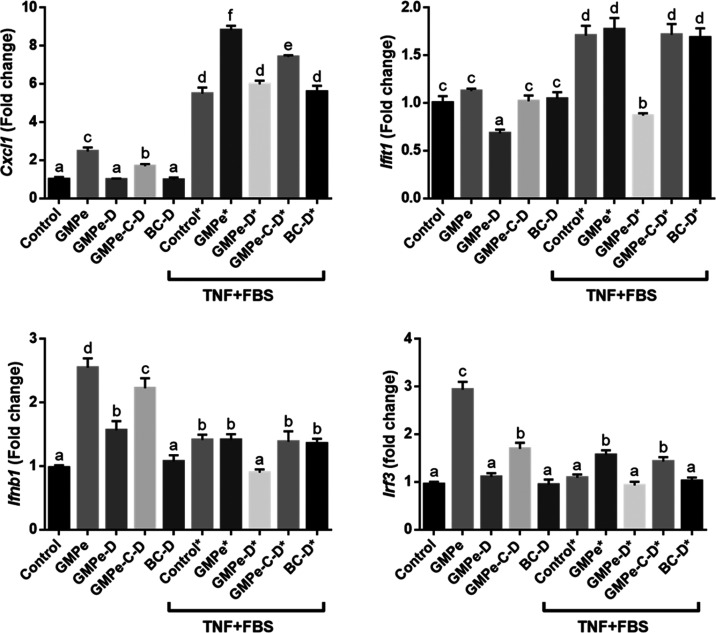
Expression of Cxcl1 and interferon-related genes in 3D mouse jejunum organoids measured by qRT-PCR. Different letters indicate different values *p* < 0.05. Data are representative of two different experiments, *n* = 4.

### 3.3 Simulated Gastrointestinal Digestion of GMPe Modifies Its Effects on Intestinal Barrier Function Genes

A completely different expression profile was observed when GMPe-D was added to 3D intestinal organoids, both under basal conditions and under TNF stimulation. Digestion and dialysis abrogated all of the effects observed for GMPe in nonstimulated organoids. In addition, and in contrast to GMPe, GMPe-D induced *Olfm4*, *Lgr5*, and *Lct*, while it only partially induced the expression of *Ifnb1* and inhibited that of *Ifit1* (Figures 5–7). When tested in TNF-stimulated organoids, a predominantly inhibitory effect of GMPe-D was observed. In fact, GMPe-D inhibited the expression of alkaline phosphatase genes (*Alpi* and *Alpl*), *Ang4*, *Ifit*, *Ifnb1*, *Lyz1*, *Muc4*, *Oas1a*, *Oas2*, *Oas3*, and *Rnasel*, and only induced that of *Muc3, Reg3g*, *Pla2g2a*, and *Olfm4*.

### 3.4 Microencapsulation of GMPe Preserves Its Main Effects on Intestinal Barrier Function Genes

GMPe was microencapsulated and subjected to *in vitro* digestion and dialysis. The resulting product (GMPe-C–D) was added to the organoid culture medium. Our results indicate that encapsulation essentially preserved GMPe bioactivity, although the magnitude of the effect on some genes was attenuated. Thus, the same level of stimulation was observed for GMPe and GMPe-C–D for *Muc3*, *Pcna*, *Oas2*, and *Pla2g2a*, while the magnitude of stimulation of *Akp3*, *Alpl*, *Axin2*, *Cxcl1*, *Ifn1b*, *Irf3*, *Pla2g2a*, *Oas2*, *Oas3*, *Reg3g*, *Rnasel*, or *Vil1* by GMPe-C–D was lower than that of GMPe. When stimulated with TNF results were quite similar. We observed that GMPe and GMPe-C–D exerted the same stimulation level for many genes while the magnitude of stimulation was lower for *Oas1*a, *Oas2a*, *Oas3*, and *RnaseI*. Only the expression of Lgr5 and Lct was higher when GMPe-C–D was added to the medium.

Empty capsules were also added to the culture medium, and interestingly, in both basal and TNF-stimulated conditions, organoids presented changes in gene expression: a higher expression of *Ly1z* and *Muc4* in the former, of *Muc4*, *Lyz1*, *Pl2g2g*, and *Reg3g* in the latter (Figure 3), indicating that this is not an inert material and can modulate gene expression itself.

## 4 Discussion

GMP has been studied as a bioactive peptide that regulates intestinal inflammation and immunity, but its specific and direct effect on intestinal epithelial cells has been poorly studied. Until recently, models of intestinal epithelial cells were restricted to established cell lines since primary mature intestinal cell types do not proliferate in culture and have limited viability. Intestinal organoids are developed from crypt cells that proliferate and differentiate *in vitro* to form 3D and 2D structures that contain all of the epithelial lineages, including goblet cells, enterocytes, enteroendocrine cells, and Paneth cells.

3D organoids are a powerful tool for functional studies involving the intestinal epithelium. Because addition of compounds to 3D organoids results in the contact with the basolateral side of epithelial cells, 2D models with open organoid monolayers have been developed. Differential effects have been shown after stimulation of the basal and apical sides of intestinal epithelial cells. Our first aim was to select a model to study the effects of GMP and both 3D organoids and a 2D monolayer model were used. We found that gene expression of 2D monolayers was not altered after GMPe addition, whereas it modulated (mainly induced) the expression of intestinal barrier genes in 3D organoids. In addition, 2D monolayers did not respond to TNF. Taken together, these data suggest that GMP is not active when in contact with the apical membrane of enterocytes or alternatively that monolayers are under-responsive to stimuli. It has been described that 3D organoids are permeable to 4 kDa dextran and partially to 10 kDa dextran. GMP molecular weight is approximately 6.7 kDa (variable depending on glycosylations), with its peptides having substantially lower molecular size.^37,38^ Thus, it is expected that cells in 3D organoids are exposed to GMP and its peptides on both the basolateral and apical sides, although not necessarily equally. Considering that most of the available studies with macro- and micronutrients use 3D organoids, we believe the 3D model constitutes a suitable model to assess differences between GMP and its peptides, and we selected it for further experiments. Although less physiological, the addition of GMP to the basolateral region would mimic situations of altered permeability. We further forced these conditions, in some experiments, by the addition of TNF to better reproduce inflammatory conditions.

The intestinal barrier is essential for the maintenance of multiple intestinal and systemic functions that, when altered, may lead to disease states.^39^ The intestinal epithelium regenerates every 5–7 days. The maintenance of the regeneration rate, which involves processes of both proliferation and differentiation, is essential for homeostasis of the barrier function. In our study, GMP induced both the expression of genes related to proliferation and stemness as well as genes related to enterocytes, mucus-producing cells, and Paneth cells, both under basal conditions and under immunological stimulation with TNF.

GMP also induced the expression of genes related to inflammation (*Cxcl1*) and defense against bacteria and viruses. CXCL1 is a chemokine that acts as a chemoattractant for several immune cells, especially neutrophils or other nonhematopoietic cells, to the site of injury or infection and plays an important role in the regulation of immune and inflammatory responses. Studies in splenocytes had previously shown immune stimulatory effects of GMP through signal transduction pathways involving the MAPK and NF-κB,^8,9^ while anti-inflammatory effects in *in vivo* models of intestinal inflammation have been described.^3–5,7^ These effects are far from incompatible, since a basal immune stimulation is needed to maintain the intestinal immune barrier,^39^ and in fact GMP does not have proinflammatory effects *in vivo*.^4^

It is very interesting to note that in our *in vitro* model GMP induced the response of various genes related to the interferon response that regulate immune response against microorganisms, including viruses but also bacteria, parasites, and fungi.^40^ This effect has not been previously described and, regarding antiviral defense, only studies indicating that GMP inhibits virus adherence have been published.^41^ Interferons strengthen the adaptive and immune cell response through direct effects and additionally impair protein synthesis, effectively inhibiting virus multiplication.^42^ IRF3 (encoded by *Irf3*) is a factor that modulates the expression of interferons in response to viruses, including IFN-β, which is encoded by the *Ifnb* gene. Type I interferons are secreted, and they have multiple actions by binding specific receptors. Among them, interferons regulate the STAT1/2 pathway and the production of the OAS and RNaseL. OAS limits viral spread by activating RnaseL, which in turn destroys cellular RNA.^43^ Our results indicate that *Irf3*, *Ifnb*, *Oas1a*, 2, and 3, and *Rnasel* were induced by GMPe, suggesting it may play a role in intestinal defense. However, further experiments will be necessary to confirm this hypothesis, involving actual infection models, which ultimately ought to also tackle the interplay with nonepithelial cells.

Since *in vivo* functions for GMP have been described, it follows that either enough GMP reaches the intestine undigested or GMP-derived peptides may exert *in vivo* effects. Our results show that intact and *in vitro* digested GMP have very different effects on intestinal organoids. The digestion of GMP abrogated the observed effects on proliferation and specific lineage cell markers. In addition, new effects were observed, like the induction in basal conditions of *Olfm4*, *Lgr5*, and *Lct*, indicating that it could induce enterocyte differentiation or cell proliferation by mechanisms that do not match those of intact GMP. The effects of GMP on immunity, including the modulation of the interferon response, were also removed by artificial digestion. Furthermore, when organoids were stimulated with TNF predominantly, an inhibitory effect of digested GMP was observed in gene expression, independent of the function studied.

Since microencapsulation protects peptides from digestion, we encapsulated GMPe and subjected GMP-enriched capsules to *in vitro* gastrointestinal digestion. Notably, encapsulated GMPe substantially preserved its effects on jejunal organoids after *in vitro* digestion, although some differences were also detected. Of note, empty capsules showed some biological effects, which could be due to the presence of P. columbina. In fact, previous reports have shown that red algae, and in particular P. columbina hydrolysates, may exert immunomodulatory effects.^44^ It is important to note that the encapsulation technique is a rapidly developing technology with a wide range of beneficial uses in dairy applications. In the dairy industry, microencapsulation is used to protect sensitive components, such as vitamins, flavors, aromas, probiotics, antioxidants, enzymes, polyphenols, and micronutrients, from adverse environments and deliver them to controlled targets. In this sense, microencapsulated ingredients can be added to various dairy products, including yoghurts, beverages, or cheese, to enhance their nutritional value or sensory properties.^45^ Taking into account the results obtained with GMPe-CD, the microencapsulated GMP could be used as a biofunctional ingredient in dairy products. However, further studies on the behavior of the microencapsulated GMP in the food matrix should be done.

In general, our data describe new effects of GMP and its derived peptides on the intestinal epithelium, suggesting that both products could be interesting functional foods. On the one hand, intact GMP modulates gene expression related to antimicrobial defense and epithelial proliferation and homeostasis. On the other hand, digested GMP has an inhibitory effect on TNF-induced immune stimulation in jejunal organoids. Although more complex, physiological, and informative than intestinal cell lines, the organoid system is devoid of extraepithelial components and therefore only allows us to draw conclusions regarding the direct effect of GMP on gene expression of intestinal epithelial cells. In addition, the use of *in vitro* digestion must be complemented in the future by *in vivo* models to confirm our results in animals.

In conclusion, here we have described in a model of intestinal organoids that GMP may contribute to the maintenance of intestinal barrier function inducing the expression of genes that are relevant for epithelial proliferation, homeostasis, and antimicrobial defense (antibacterial peptides, type I interferon genes, and *Cxcl1* cytokine). These effects depend upon the presence of intact GMP, as they were dramatically altered by *in vitro* digestion and were preserved by encapsulation. Peptides derived from GMP showed interesting effects inhibiting TNF-induced immune stimulation. Our results are an important contribution to describe the functionality of both intact and digested GMP at the intestinal epithelium level. It would be interesting to develop methodologies that allow the encapsulation of the peptide in order to preserve its effects after oral administration *in vivo*. In this regard, we have developed an encapsulation procedure that is effective in maintaining the peptide and its activity under simulated digestion conditions. Although organoids are an important advance for *in vitro* studies, there are obvious limitations including, for example, the lack of lamina propria cells or connections with the nervous system; therefore, *in vivo* studies to strengthen the translation relevance of our study should be carried out.

## Supplementary Material



## Notes

Primers used in the RT-qPCR analysis (PDF)
