# Supplementary material for: Casein Glycomacropeptide Regulates Gene Expression in Intestinal Epithelial Cells: Effect of Simulated Gastrointestinal Digestion and Peptide Microencapsulation
Source: J Agric Food Chem. 2025 Feb 8;73(7):4105–15. doi: 10.1021/acs.jafc.4c10146 (PMC12150316; doi:10.1021/acs.jafc.4c10146)
Supplement: Supplementary file 1 [file jf4c10146_si_001.pdf]

Table S1. Primers used in the RT-qPCR analysis

Gene	Forward 5'-3'	Reverse 3'-5'
18s	TGGTGGAGCGATTTGTCTGG	ACGCTGAGCCAGTCAGTGTACG
Ang4	TTTGGAATCACTGTTGGAAG	TGCTGACGTAGGAATTTTTC
Alpi	CATGGACATTGATGTGATCC	AGACTGGTTACTGTCACTTG
Alpl	CTGCCACTGCCTACTTGTGT	GATGGATGTGACCTCATTGC
Ascl2	ACTGTTGTAGGACTCTACTG	ATAGGCTTCAGAAGAGGA
Axin2	AAGATCACAAAGAGCCAAAG	GAAAAAGTAGGTGACAACCAG
Cxcl1	CCGAAGTCATAGCCACACTCAAG	ACCAGACAGGTGCCATCAGAG
Hprt	AGGGATTTGAATCACGTTTG	TTTACTGGCAACATCAACAG
Ifit1	AGAACAGCTACCACCTTTAC	TTCTTGATGTCAAGGAACTG
Ifng	CTAGCTCTGAGACAATGAAC	CTCTTTTCTTCCACATCTATGC
Ifr3	CTCAGCTGCTACCAATAAAG	CTTGTAGAATAACCACCAGC
Lct	TTCCTATCAGGTTGAAGGTG	GTCATTCCCAATCTTCAGTG
Lgr5	AGAACACTGACTTTGAATGG	CACTTGGAGATTAGGTAACTG
Lyz1	CAAGATCTAAGAATGCCTGTG	TTCCGAATATACTGGGACAG
Muc3	AAAGATTACCTCCCATCTCC	TAAAACTAAGCATGCCCTTG
Muc4	GGATTCCTTCTACGTTACAG	GTAGAGAAATCAGCATCAG
Oas1a	ATTAAAAAGGATGGTTCCCG	ATGTCCAGTTCTCTTCTACC
Oas2	CTGGTACAAACAGTATGAAAGG	GTAATTGACTGTCCAGAAGATG
Oas3	CCAACTTAAGAGCCTGATG	GCCTCTCCTCCTTTATATCG
Olfm4	CGAAGTGGAGATAAGGAATATG	CTCACATTCTTTCAGCTTGG

Pcna	CTGAGGTACCTGAACTTTTTC	TATACTCTACAACAAGGGGC
Pla2g2a	CAAGAAACCATACCACCATC	TCTTAAGCCGAATCATTTCC
Ppib	CAAATCCTTTCTCTCCTGTAG	TGGAGATGAATCTGTAGGAC
Reg3g	CAGAGGTGGATGGGAGTGGAG	CACAGTGATTGCCTGAGGAAGAG
RNAsel	ATACTGTAGGTGATCTGCTG	AAGTATCTCCTTCATTCCCC
Sis	GAAGATAACTCTGGCAAGTC	GTCCAATGAGCTCTTGATATTG
Villina	TATGGAGGATCGAGGCTATG	AGGTAGTCATCCATCTGTGT

2
